# Recent Progress of Natural and Recombinant Phycobiliproteins as Fluorescent Probes

**DOI:** 10.3390/md21110572

**Published:** 2023-10-31

**Authors:** Huaxin Chen, Jinglong Deng, Longqi Li, Zhe Liu, Shengjie Sun, Peng Xiong

**Affiliations:** School of Life Sciences and Medicine, Shandong University of Technology, Zibo 255000, Chinaxiongp@sdut.edu.cn (P.X.)

**Keywords:** phycobiliprotein, fluorescent probe, algae, phycobilin

## Abstract

Phycobiliproteins (PBPs) are natural water-soluble pigment proteins, which constitute light-collecting antennae, and function in algae photosynthesis, existing in cyanobacteria, red algae, and cryptomonads. They are special pigment–protein complexes in algae with a unique structure and function. According to their spectral properties, PBPs can be mainly divided into three types: allophycocyanin, phycocyanin, and PE. At present, there are two main sources of PBPs: one is natural PBPs extracted from algae and the other way is recombinant PBPs which are produced in engineered microorganisms. The covalent connection between PBP and streptavidin was realized by gene fusion. The bridge cascade reaction not only improved the sensitivity of PBP as a fluorescent probe but also saved the preparation time of the probe, which expands the application range of PBPs as fluorescent probes. In addition to its function as a light-collecting antenna in photosynthesis, PBPs also have the functions of biological detection, ion detection, and fluorescence imaging. Notably, increasing studies have designed novel PBP-based far-red fluorescent proteins, which enable the tracking of gene expression and cell fate.

## 1. Introduction

**Figure 1:**
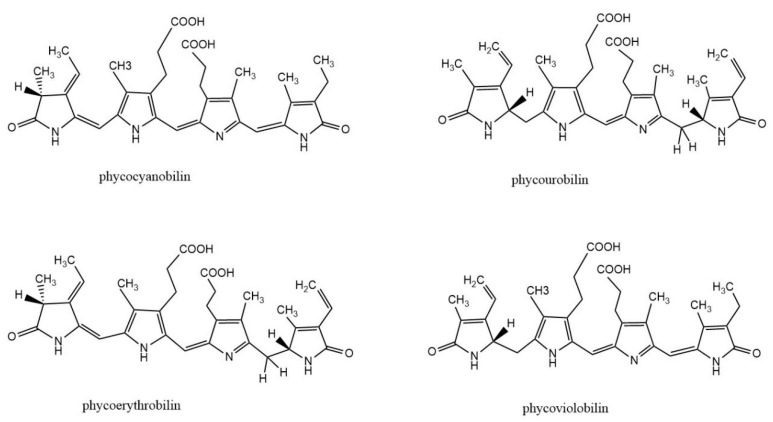
Structure of biliverdin and four phycobilins: phycocyanobilin, phycourobilin, phycoerythrobilin, and phycoviobilin.

Phycobiliprotein (PBP) is a kind of natural water-soluble pigment protein, which is a light-harvesting antenna in algae photosynthesis. It is commonly found in cyanobacteria, red algae, and cryptomonads. Most PBPs exist in the form of phycobilisomes and are distributed on the thylakoid membrane of algae. The basic building block of PBS is monomers, which are heterodimers of two subunits (α and β). Each subunit of PBPs carries one to three linear tetrapyrrole chromophores (phycobilins) at specific cysteine residues. Phycobilin is a kind of linear chain opening tetrapyrrole ring compound. There are four types of phycobilins: phycocyanobilin (PCB), phycourobilin (PUB), phycoerythrobilin (PEB), and phycoviolobilin (PXB) (Figure 1). Based on their spectral properties, PBPs are mainly divided into four categories: allophycocyanin (APC), phycoerythrin (PE), phycocyanin (PC), and phycoerythrocyanin (PEC). According to the energy level related to light absorption, PBPs can be divided into three types: high energy (PE and PEC), medium energy (PC), and low energy (APC). The absorption and fluorescence emission spectra of PBPs with different energy levels overlap strongly.

Environmental factors such as the temperature, solution pH, light intensity, and ionic intensity will affect the stability and fluorescence characteristics of PBPs [1]. The aggregation state of PBPs is related to their concentration, pH, and ions in solution [2]. There is a dynamic equilibrium between different aggregation states of PBPs in solution. In the purification process, the dissociation of (αβ)_3_ and (αβ)_6_ units usually leads to the blue shift of fluorescence peaks [3]. Generally, the environmental temperature of PBPs is not more than 65 °C and a higher temperature destroys the protein structure, leading to denaturation and inactivation [4]. In addition, high concentrations of metal ions and organic solvents will also destroy the hydrogen bond and secondary bond of PBPs, resulting in inactivating and precipitation.

The purification process of natural PBPs is complicated and laborious [5,6]. In general, PBPs’ purification involves two steps. The initial step is the extraction of PBPs from algal cells. Different methods are adopted to disrupt algal cells, including chemical treatment, physical treatment, and enzymatic treatment. The second step is the purification of PBPs from crude extractions by multiple separation processes including ammonium sulfate precipitation, chromatography, membrane filtration, or two-phase aqueous extraction. In recent years, new strategies for producing recombinant PBPs in heterologous hosts by genetic engineering have been developed (Table 1). Affinity and biospecific recognition tags can be added to PBPs, facilitating the purification and labeling of PBPs. PBPs have unique spectral properties and biological activities and can be used as natural pigment dyes [7], antioxidants [8], fluorescent probes [9], and photosensitizers [10]. The far-red fluorescent protein smURFP, evolved from the α subunit of APC, has shown great advantages in deep imaging and labeling of mammalian tissues. Far-red fluorescent protein is an ideal choice for in vivo imaging because endogenous biomolecules have less scattering, absorption, and secondary emission of its emitted light. SmURFP is covalently linked to BV without the participation of lyase, with an excitation/emission peak of 642/670 nm, a large excitation coefficient (180,000 m^−1^ cm^−1^), quantum yield (18%), and comparable photostability to eGFP. SmURFP has significantly increased BV incorporation rates and protein stability compared to the bacteriophytochrome (BPH) FPs.

**Figure 2:**
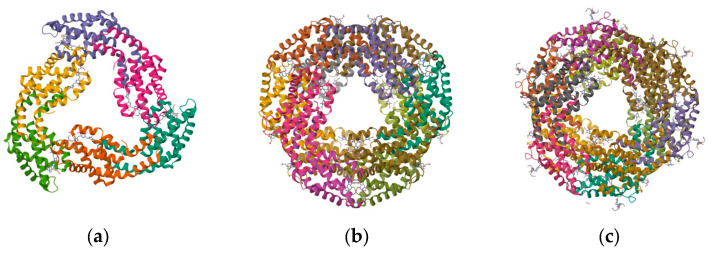
Structure of PBPs. (**a**) Structure of APC from marine *Cyanobacteria Phormidium* sp. A09DM; (**b**) structure of C-PC of *Thermosynechococcus vulcanus* at 2.5 Angstroms; and (**c**) structure of C-PE from marine *Cyanobacteria Phormidium* sp. A09DM.

In this paper, we describe the structure (Figure 2), conjugation, and labeling of PBPs and their application as fluorescent probes in biological and iron detection and cell imaging. An emphasis is paid to the progress of recombinant PBPs with respect to their biosynthesis, fusion expression, and directed evolution. Finally, we discuss the key issue and future prospective for the further exploration of these valuable proteins.

## 2. Natural PBPs

PBPs are suitable for applications that require either high sensitivity or simultaneous multicolor detection. They share some advantages over commonly used organic dyes: (1) relatively large Stoke shift; (2) high absorbance coefficient and fluorescence quantum yield; (3) fluorescence not quenched by external agents; and (4) high water solubility and a number of sites for conjugation to many biological and synthetic molecules.

### 2.1. APC

APC is located in the core of phycobilisome in the form of a cylinder, which exists in all organisms containing PBPs. The APC αβ monomers in the core cylinders consist of ApcA, ApcB, ApcD, and ApcF and are divided into an APC, APC-B, and APC core-membrane linker (APC-Lcm). The latter participates in forming the basic cylinder [11,12,13]. APC-B occupies a position between APC and chlorophyll a in the energy transfer pathway from the accessory pigments to chlorophyll a in species [14]. APC receives light energy absorbed from PC and its maximum absorption peak is at 650 nm [15].

APC is composed of αβ subunits with each subunit carrying a PCB chromophore. The interactions between PCBs bound to α and β subunits of adjacent protomers in trimeric APC complexes are responsible for a large bathochromic shift of about 20 nm. At the same time, this interaction contributes to the notable sharpening of the long-wavelength absorbance band. The structure of APC from different sources is highly conserved. Due to the lack of the stabilizing effect of the γ subunit, subunits are dissociated when APC is highly diluted. Consequently, the absorbance maximum for APC shifts from 650 nm to 618 nm and the fluorescence maximum shifts from 660 nm to 650 nm. Such dissociation of APC can be avoided by the crosslinking of α and β subunits using chemical agents. The spectral characteristics and structure of the cross-linked APC are similar to those of the untreated APC [11].

### 2.2. PC

PC is mainly found in cyanobacteria, red algae, glaucophytes, and some cryptophytes [16]. Based on their spectral characteristics, PC can be divided into C-PC and R-PC [17]. According to differences in PBPs with specific light spectra, R-PC can be divided into R-PC I and II as well as other types [18,19]. For R-PC, an α subunit contains one PCB but a β subunit carries one PCB and one PEB. C-PC usually shows a one-peak absorption spectrum with a peak within 610–620 nm whereas R-PC has a double-peak absorption spectrum around 550 nm from PEB and the second at 615 nm from PCB [20]. When isolated from phycobilisome, PC exists as a hexameric structure (αβ)_6_ at pH 5.0–6.0 and a trimeric structure (αβ)_3_ at pH 7.0. The separation process of C-PC is complex, including cell disruption, primary extraction, and purification. Some processes require large equipment and long processing times, resulting in very low yields of C-PC [21]. C-PC can be extracted from wet or dry biomass, with higher yields from the latter. However, the high temperature used during the drying process not only affects the phycobilisome status but also results in the loss of C-PC [22].

### 2.3. PE

PEs are the most abundant PBPs in many red algae and in some unicellular cyanobacteria. They are generally classified into three types based on their spectral properties: B-phycoerythrin (B-PE), C-phycoerythrin (C-PE), and R-phycoerythrin (R-PE) from Bangiophyceae (primitive filamentous Rhodophyta), cyanobacteria, and complex Rhodophyta, respectively [13,23]. PE is a hexamer discoid complex composed of three monomers. A monomer (αβ) is composed of two subunits by electrostatic interaction and then covalently connects with a γ subunit to form trimers. Two trimers are recombined face to face to form a more stable hollow hexamer (αβ)_6_ γ. The molecular weight of subunit α is about 13~20 kDa and the β subunit is higher than that of subunit α, which is about 14~24 kDa. The molecular weight of subunit γ is about 30~34 kDa. Due to the presence of the γ subunit, PE is more stable than other PBPs [24].

**Figure 3:**
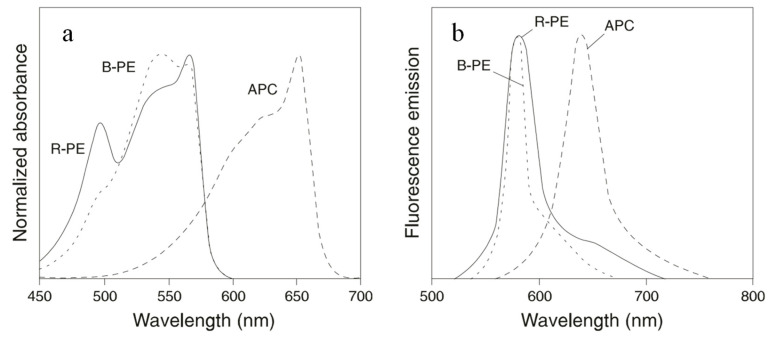
Normalized absorbance spectra (**a**) and fluorescence spectra (**b**) of B-PE, R-PE, and APC.

B-PE and R-PE are the most widely used PBPs in cell sorting and cell analyses. Each hexamer of the proteins carries 34 phycobilins. They absorb light between 480 nm and 570 nm and emit fluorescence maximally at 580 nm. B-PE carries 32 PEB and 2 PUB molecules. This protein has absorption maxima at 540 nm and 567 nm, with a shoulder at 490 nm. R-PE contains 25 PEBs and 9 PUBs. Therefore, R-PE absorbs light maximally at 490 nm and 567 nm (Figure 3). On a molar basis, PE has a fluorescence intensity equivalent to about 30 fluorescein or 100 rhodamine molecules at comparable wavelengths. The broad excitation spectra of PE allow simultaneous detection of cells by immunofluorescence analysis or sorting by flow cytometry using a single excitation laser line.

### 2.4. Phycobiliprotein Conjugates

Scattering and autofluorescence from the biological system increase the background at the detection wavelength and thus decrease the detection limit in fluorescence assays. Autofluorescence arising from components such as porphyrins and flavins is characterized by a small Stokes shift. Fluorescent probes with large Stokes shifts are advantageous since the fluorescence can be detected at longer wavelengths where most autofluorescence is minimal. In addition, these probes allow simultaneous multicolor labeling and detection of several targets with excitation by a single excitation source.

In order to create fluorescent dyes with large Stokes shifts, researchers have developed fluorescent tandem PBP conjugates. The PBP conjugates can be prepared by covalent attachment of a PBP to another PBP or organic dye. As long as only a small number (three to six) of PBP side-chains are modified in each molecule, the physical or spectral properties of the PBP are almost unchanged [5]. A number of PBP conjugates have been prepared. These novel fluorescent tandem dyes have significantly increased the number of parameters that can be measured simultaneously at the single-cell level. They can also be coupled with commercial monoclonal antibodies to expand the capacity of the general flow cytometry platform [25].

#### 2.4.1. Crosslinkage of PBPs

An attractive feature of serial PBP conjugates is that several dyes with different emission spectra can be excited efficiently with a single excitation wavelength. This means that two or more components can be analyzed simultaneously with a single excitation beam. With the help of intermolecular energy transfer, the Stokes shift can be greatly increased so that the conjugates can emit fluorescence with longer wavelengths. In fluorescence-activated cell sorting, fluorescence microscopy, and fluorescence immunoassay analyses, tandem PBP conjugates are very commonly used. PE is a particularly suitable energy donor because it has a broad and strong absorption spectrum. APC is a suitable energy acceptor since its emission peak at 660 nm is far from the absorption range of PE donors. Due to excellent spectral overlap between the donor and the acceptor, the energy transfer from PE to covalently linked APC was very effective. Glazer and Stryer showed that in a B-PE-APC conjugate, the energy absorbed by PE was transferred with an efficiency of 90% to APC. Excitation of the conjugate at 488 nm led to maximum fluorescence emission at 660 nm, giving a Stokes shift of 172 nm [26]. The chromophores in PE were coupled by resonance interaction so that the excitation could be quickly transferred to the specific chromophores near the interface with APC. This energy transfer was enhanced by the increase in APC chromophores in contact with PE. The absorption spectrum of PE and APC conjugate is the sum of the contributions of each component. In the absence of reducing agent, the aliphatic disulfide bridge between PE and APC was stable. This disulfide bond can be cracked by adding a high concentration of dithiothreitol (DTT).

#### 2.4.2. Crosslinkage of PBPs and Dyes

In order to reduce the influence of autofluorescence, researchers have developed some red and far-red fluorescent dyes to emit fluorescence with wavelengths greater than 600 nm. Waggoner and his colleagues developed the fluorescent molecules cyanine 5 (Cy5) and cyanine 7 (Cy7) [27,28]. These fluorescent dyes have good characteristics: (1) a very high extinction coefficient, (2) high quantum efficiency, and (3) fluorescence emission in the red (Cy5) and far red (Cy7) region. With the help of these exceptional spectral characteristics, they are very bright in immunofluorescence applications. In addition, Cy5 and Cy7 have relatively narrow excitation and emission bands, which only slightly overlap with other fluorescent agents. This means that they can be used together with several other fluorescent agents. The energy transfer pathway between PBPs and organic dyes reduces photobleaching and triplet-state conversion to some extent. It can improve the light resistance of PBPs [29]. It has been shown that conjugates containing three molecules (or more) of Cy5.18 per PE hexamer proceeds with an efficiency exceeding 90% and an emission maximum at 667 nm [27].

When Cy5 is covalently linked to PE forming a tandem dye (Cy5PE), the donor PE can be excited with 488 nm light and the fluorescence emitted by the receptor Cy5 is detected at 670 nm through energy resonance energy transfer (FRET) [30,31,32]. FRET tandem dyes are suitable for flow cytometry and other immunofluorescence applications. Cy7 can be conjugated to PE (Cy7PE) and excited at 488 nm; it emits fluorescence at 780 nm, resulting in a Stokes shift of about 300 nm. These three kinds of fluorescent agent are commonly used for three-color immunofluorescence analysis by single-laser flow cytometers.

Cy7 can be conjugated to APC (Cy7APC) and the tandem can be excited at 600–647 nm and emitted at 780 nm [33]. Consistent conjugations with Cy7 require that the PBP and the Cy7 are at the same concentration. The general method is to use SPDP to directly react with PBP and then conjugate the Cy7PBP tandem with the heterobifunctional linking reagent SMCC. Generally speaking, with the increase in the ratio of Cy7 to PBP, the energy transfer efficiency can be improved but the potential for self-quenching becomes greater. When the ratio of Cy7 to PBP increased above a certain point, the total Cy7 fluorescence begins to decrease, which indicates that self-quenching occurs significantly.

Recently, Seong et al. [25] engineered six new fluorescent dyes which emit fluorescence at the near-infrared region: PE-750, PE-800, and PE-830 for the 488 nm laser and APC-750, APC-800, and APC-830 for the 640 nm laser. These novel fluorescent pigments are named after as a combination of donor molecules (PE and APC) and their peak emission wavelengths. They were created by covalently linking a PBP to small organic molecule acceptor. The flow cytometer based on photomultiplier (PMT) was compared with the flow cytometer based on Avalanche Photodiode (APD) to evaluate the sensitivity of these novel antibody-conjugated fluorochromes in the near-infrared region. The results showed that APD had better sensitivity than PMT when the emission wavelength exceeded 800 nm. These novel fluorescent dyes can be coupled with commercial monoclonal antibodies using click chemical reactions between methyl-tetrazine and trans-cyclooctene-tetrazine. These novel antibody-conjugated fluorochromes can be easily combined with other commercially available fluorescent dyes. A stability test with fixative/permeabilization buffers revealed acceptable brightness and stability when these fluorochromes were conjugated to monoclonal antibodies.

### 2.5. Labeling of PBPs

Use of the exceptional fluorescence of PBPs generally requires chemical coupling of PBPs to antibodies. The ways of binding PBPs with antibodies are divided into direct labeling and indirect labeling. Due to the molecular weight of PBP being larger than that of traditional fluorescent dyes, it is easy to cause steric hindrance. In addition, a crosslinking reaction may alter the structure and activities of PBPs and antibodies. Light, pH, temperature, and the concentration and type of crosslinking agents affect the PBP conjugates. Some earlier methods for preparing PBP conjugates with antibodies yield a reductively unstable disulfide linkage. It is recommended that the conjugates are prepared by using agents that yield a more stable thioether protein–protein linkage.

#### 2.5.1. Direct Labeling

Direct labeling uses the amino, imino, sulfhydryl, and other active groups of PBPs to covalently cross-link with the primary antibody. Protein cross-linking reagents are divided into intramolecular homo-bifunctional reagents and intermolecular hetero-bifunctional reagents. Previous studies showed that the labeling efficiency of homo-bifunctional reagent is not ideal, leading to the formation of different polymers [34]. Intermolecular hetero-bifunctional reagents, such as SPDP and SMCC, are often used for direct labeling. SPDP can be used to cross-link PBPs and antibody proteins through amino groups without forming polymers of PBPs or antibody proteins themselves.

**Figure 4:**
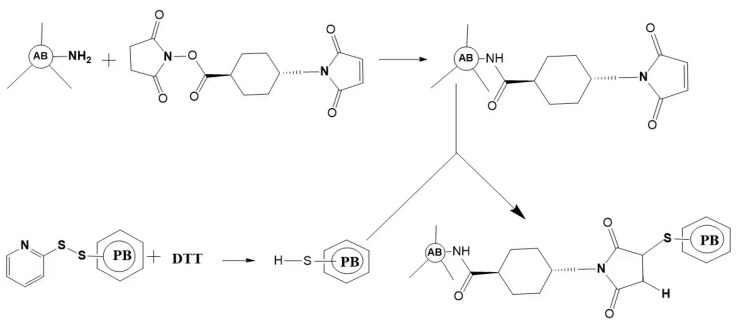
Schematic outlining of the crosslink of a phycobiliprotein to an antibody. The antibody (AB) is treated at pH 7.5 with succinimidyl trans-4-(maleimidylmethyl) cyclohexane-1-carboxylate (SMCC). This converts some lysine residues of the antibody to thiol-reactive maleimides. Thiolated phycobiliprotein (HS-PB) is prepared by a reduction in the appropriate succinimidyl 3-(2-pyridyldithio) propionate (SPDP) modified phycobiliprotein with dithiothreitol (DTT). After dialysis, the above two protein conjugates are mixed to yield a stable thioether crosslink.

Direct labeling is easy to operate. Due to the requirement of labeling only once, the interference caused by the non-specific absorption of antibodies and cells is minimal. However, the direct labeling method has some shortcomings, such as the need to label analytical reagents one by one when multi-component simultaneous detection or multi-color cell labeling is needed. In addition, it is easy to produce steric hindrance because of the high molecular weight of PBPs (Figure 4).

#### 2.5.2. Indirect Labeling

Indirect labeling means that PBPs do not directly label primary antibody molecules but use a second reagent as a bridge to couple PBPs with antibody molecules. Compared with the direct method, the indirect method has the advantages of high detection sensitivity, saving both cost and tedious labor.

The most commonly used indirect labeling method is the biotin–avidin system method. Avidin is an alkaline tetrameric glycoprotein. Each subunit binds to biotin with extremely high affinity. With the help of the interaction between biotin and avidin, the biotinylated PBPs and antibodies are connected. Antibodies specifically attach to antigens and thus an antigen–antibody–biotin–avidin–biotin–PBPs system is formed. This bridge system offers some advantages, including improvement of the sensitivity of fluorescent probes and an increase in the immune response rate by avoiding the steric hindrance of macromolecular PBPs.

**Figure 5:**
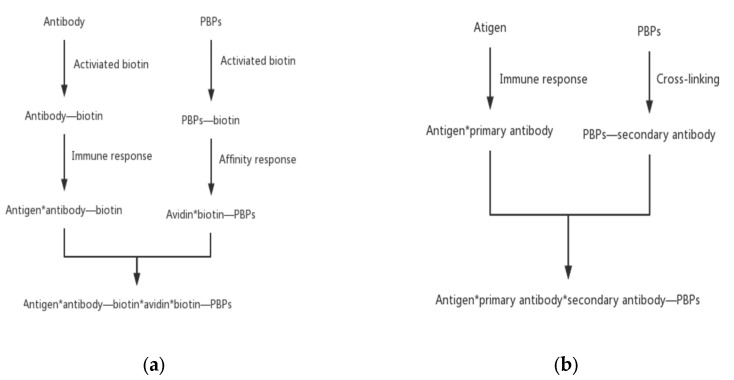
Indirect PBPs labeling methods. (**a**) The biotin–avidin system method and (**b**) the secondary antibody method.

Another type of indirect labeling method is called the secondary antibody method. This method is similar to direct labeling except that PBPs label the secondary antibody instead of the primary antibody. PBPs bind specifically with the secondary antibody to form a secondary antibody–primary antibody–antigen complex. With this method, only one PBP-labelled antibody against the primary antibodies needs to be prepared. This method can reduce the time of finding specific antibodies and avoid the tedious preparation of PBP-labeled antibodies one by one (Figure 5).

## 3. Recombinant PBPs

In addition to the extraction and purification of natural PBPs from algae, heterologous biosynthesis of PBPs by engineered microorganisms offers an attractive option. With progress in recombinant DNA technology, the complete pathway of PBPs has been successfully constructed in engineered heterologous hosts. The biosynthesis of PBPs involves two processes: (1) the expression of apo-protein, lyases, and the enzymes responsible for the biosynthesis of phycobilins and (2) the attachment of phycobilins to apo-protein mediated by specific lyases. When bacteria are engineered to produce recombinant PBPs, special molecular tags are often used. These tags facilitate the purification of the recombinant PBPs [35,36,37,38]. The biosynthesis of phycobilins comes from the metabolism of heme. Heme is split into biliverdin (BV) by heme oxygenase (HO1) and then BV can be reduced by ferredoxin-dependent bilin reductases. The attachment of phycobilins to apo-protein can be catalyzed by autocatalysis or specific lyases. A previous study showed that PCB can spontaneously form pigment–protein structures in vitro which have similar spectral characteristics to natural proteins [37]. However, the attachment of phycobilin to apo-PBP is more efficient and accurate under the catalysis of lyases. Therefore, the involvement of PBP lyases is key to the efficient synthesis of recombinant PBPs. An individual subunit of α or β is easier to prepare from engineered hosts than natural PBPs and may offer some advantages due to its small size.

PBP lyases are less specific to phycobilins in *E. coli* compared to their native host. A variety of PBPs carrying noncognate phycobilins have been produced in engineered *E. coli*. In our previous study, holo-ApcA carrying PEB was successfully reconstituted in *E. coli*. This recombinant protein showed a distinct spectral property from the native holo-ApcA carry PCB. Notably, holo-ApcA carrying PEB has a much higher quantum yield than the native protein. In sandwich immunoassay fluorescence assay, we showed that the detection limit for AFP detection by SLA–PEB and SLA–PCB were 0.11 and 0.35 ng/mL, indicating superior performance of recombinant PBP with noncognate phycobilin [39].

The main shortage of recombinant PBPs is the lack of full chromophorylation of PBPs. Previous studies showed that the percentage of chromophorylation for recombinant PBP produced in *E. coli* varied from 17.4% to 71.9% [40]. For most recombinant PBPs, the genes involved in their biosynthesis are expressed in *E. coli* with multiple plasmids. The imbalance of gene expression and/or plasmid loss (gene loss) are the main reasons for the inefficiency of PBP chromophorylation. Strategies using a single plasmid containing the full PBP pathway genes in *E. coli* may overcome gene loss during cell division and the addition of many antibiotics [41].

Most recombinant PBPs are produced in *E. coli*. By comparison, fewer are reconstituted in eukaryotic hosts. Industrial microbes such as Saccharomyces cerevisiae and Pichia pastoris could be good alternatives for the production of PBPs. The development of genetic editing tools such as CRISPR will facilitate the construction of engineered strains for the mass production of fluorescent PBPs.

### 3.1. Recombinant PBPs Equipped with a Strep2 Tag

A Strep-tag II (WSHPQFEK) is a short peptide tag obtained by genetic engineering of a Strep-tag (WRHPQFGG), which has a strong affinity to the Strep-Tactin mutant with a K_d_ value close to 10^−6^ M [42,43]. Compared with the conventional Strep-tag, Strep-tag II has a significant advantage. It can be fused at either the N-terminal or C-terminal end of the target protein, overcoming the disadvantage that Strep-tag can only be fused at the C-terminal end of the target protein. This tag can enhance the binding ability to Strep-Tactin by sequence repetition. The fusion of the Strep-tag II tag with the streptavidin affinity column has the remarkable feature of mild elution conditions and the target protein can be recovered by using a low concentration of desulfated biotin. A particular benefit of Strep-tag II is its rather small size and the fact that it is almost biochemically inert. Therefore, protein folding or secretion is not affected and does not generally interfere with protein function. Strep-tag II is particularly suitable for the analysis of functional proteins because the purification process can be maintained under physiological conditions. This not only allows the isolation of sensitive proteins in their natural state but also the purification of intact protein complexes, even if only one subunit carries the tag.

Cai constructed a specific cloning vector containing a Strep2 tag that binds to streptavidin and successfully expressed it in the cyanobacterium *Anabaena* sp. PC7120. This tagged α or β subunit forms a complex with the appropriate wild-type subunit in vivo. The results showed that the spectral properties of this modified construct were highly similar to those of unmodified PC. All of these constructs bind in vivo to the rod-like substructure of the light-trapping complex PC. In such recombinant PC equipped with a stable trimerization domain, >75% of the fusion protein binds specifically to chain streptavidin-coated beads. Thus, this method provides a feasible approach for the construction of oligomeric PBP complexes equipped with affinity purification tags and biospecific recognition structural domains that can be used as fluorescent probes [44].

### 3.2. Recombinant PBPs Fusion with Streptavidin

Due to the presence of polysaccharide in the structure of avidin, other affinity reactions will occur and interfere with the labeling of PBPs. At present, researchers gradually use streptavidin, whose structure is similar to avidin, to interact with biotin. Streptavidin has many active groups and exists in the form of a tetramer and can bind four biotin molecules [45]. The affinity of streptavidin with biotin is extremely high. The Kd value is up to 10^−13^ M, an important factor for using streptavidin as an alternative choice. The specific sequence of target miRNAs can be detected by binding PE with streptavidin and then linked with biotin. The detection range is from 2.5 pM to 1.25 nM and the detection limit is 2 pM [46].

By using recombinant PCR, an artificial synthesized streptavidin gene was fused with an ApcA gene (denoted as SLA). The fusion protein contains two functional domains, streptavidin and holo-ApcA, with a 26 AA peptide linker to ensure the activity of each functional domain. SLA–PCB was produced by using dual plasmid expression systems in *E. coli*, containing fusion genes of SLA and lyase CpcS and plasmid pRSF-Ho1-PcyA containing Ho1 and PcyA genes. SLA–PEB was produced by dual plasmid expression systems containing pCDF-SLA-cpcS and pRSF-Ho1-pebS containing Ho1 and PebS genes. Both SLA–PCB and SLA–PEB can be used in a fluorescence sandwich immunoassay [47]. Sodium dodecyl sulfate-polyacrylamide gel electrophoresis (SDS-PAGE) analysis indicated that the fusion protein existed in the form of tetramer. The biotin binding activity of SLA–PEB was similar to that of recombinant streptavidin while the biotin affinity activity of SLA–PCB was slightly lower than that of recombinant streptavidin [39]. 

The researchers expressed streptavidin–phycocyanin α subunit–phycocyanobilin (SA–PCA–PCB) and streptavidin–phycocyanin α subunit–phycoerythrin (SA–PCA–PEB) fusion proteins in *E. coli*. The dual-functional SA–PBPs were used to detect α-fetoprotein (AFP) and carcinoembryonic antigen (CEA) using the sandwich enzyme-linked immunosorbent assay (ELISA) method and the results showed a good linear relationship between SA–PCA–PCB and SA–PCA–PEB within a concentration range of 0–50 ng/mL. These probes can be used not only for the diagnosis of early-stage liver cancer (such as AFP and CEA) but also for a variety of other immunoassays by simply changing the antigens and cognate antibodies, showing high expandability and compatibility as well as the advantages of simplicity, low cost, and time-saving [48]. Natural PE requires many extraction steps, a high cost for separation and purification, and needs to be covalently cross-linked with streptavidin molecules for detection purposes [49]. The expression of recombinant streptavidin–PBP fusion protein is simple to prepare and it can serve as an alternative to natural PE. It will have a wide range of applications in the future with its simple preparation and low cost.

## 4. Far-Red Fluorescent Proteins Evolved from PBPs

A longer wavelength emission would provide greater tissue penetration. Many far-red fluorescent proteins (FPs) with autocatalytic function are derived from the mutant orange fluorescent protein or red fluorescent protein tetramer. To meet the needs of deep tissue imaging, a series of new far-red fluorescent proteins have been developed. Currently, the common FPs include monomer mNeptune and TagRFP657; dimerized eqFP650 and eqFP670; and tetrameric E2-Crimson [50,51,52,53]. FPs are the ideal choice for in vivo imaging because their excitation wavelength can reduce light scattering and auto-fluorescence [54]. FPs can track gene expression, the cell cycle, and the expression of fusion proteins [55,56,57,58]. The maximum excitation wavelength of jellyfish/coral FPs is less than 610 nm and requires O_2_. When chromophores are formed, hydrogen peroxide (H_2_O_2_) will be produced so the expression system needs to be tolerant of reactive oxygen species [59]. H_2_O_2_ affects cell survival, growth, and differentiation, which may complicate experimental results because the production of H_2_O_2_ could alter the immune system/inflammation response and/or alter disease progression [60,61,62,63]. It is urgent to find fluorescent proteins that can bind endogenous luminescent chromophores to eliminate hydrogen peroxide production.

A new fluorescent protein with 20 mutation sites was evolved from the APCα from *Trichodesmium erythraeum* (TeAPCα) which was named a small ultra-red fluorescent protein (smURFP) [64]. SmURFP fluorescence is visible without exogenous BV and may be beneficial for imaging cancer/maladies in vivo. It was covalently linked to BV without lyase, with an excitation/emission peak of 642/670 nm. SmURFP showed a homodimer of 32 kDa on a natural gel. The tandem dimer smURFP (TDsmURFP) was produced by adding a linker of 23 amino acids between subunits and its fluorescence in bacteria was about 70% of smURFP. Wu et al. [65] expressed smURFP in large quantities in *E. coli* using genetic engineering technology; purified the protein by affinity chromatography, ion exchange chromatography, and gel filtration chromatography; and obtained high-quality smURFP protein crystals through screening and optimizing the crystallization conditions. The three-dimensional spatial structure of smURFP crystals and the complex crystals of smURFP and BV were analyzed to reveal the fluorescence mechanism of the protein. APCαC52 is conserved and covalently attaches BV. SmURFP fluorescence requires BV binding and covalent attachment. Therefore, the supply of BV limits the brightness of smURFP fluorescence, but with the extension of half-life to 39 min, the fluorescence approaches its gradual level regardless of the BV concentration. BV availability is limited by the permeability of the cell membrane. SmURFP can use BVMe2 as a chromophore because it is best at penetrating membranes. Compared to 25 μm BV, the fluorescence of smURFP with BVMe2/PCB (3 h) significantly increased by 18 times and 10 times, respectively.

BeiDou fluorescent proteins (BDFPs) are derived from the APC β subunit ApcF2 which is induced by far-red light. They can covalently bind to BV to produce far-red light and near-infrared fluorescence. The maximum emission wavelength for far-red BDFPs is 670 nm and that of near-infrared BDFPs is 710 nm. BDFP1.1 is a fluorescent protein with a fluorescence peak of 710 nm and covalently binds to BV. BDFP3.1, which is obtained through molecular evolution based on a core-membrane linker, LCM (ApcE2), and non-covalently binds to phytochromobillin (PФB). BDFP3.3 is a red fluorescent protein derived from BDFP3 and it binds to PEB non-covalently, with an absorption peak of 608 nm and a fluorescence peak of 619 nm.

Hou et al. [66] designed three trimeric fusion proteins: BDFP1.1:3.1:1.1, BDFP1.2:3.3:1.2, and BDFP1.6:3.3:1.6. The emission wavelength of BDFP1.1:3.1:1.1 is 722 nm, existing as a monomer. The emission wavelengths of the latter two trimeric fusion proteins are 670 nm and they show a high fluorescence brightness when expressed in mammalian cells existing as monomers. BDFP1.1:3.1:1.1 effectively combines the fluorescence brightness of BDFP1.1-BV and the red-shifted spectrum of BDFP3.1. The energy of BDFP1.1-BV (as the donor) is transferred to BDFP3.1 (as the acceptor) through FRET so as to improve the fluorescence brightness at 720 nm. BDFP1.2:3.3:1.2 and BDFP1.6:3.3:1.6 also use FRET to transfer energy from BDFP3.3 (as the donor) to BDFP1.2-BV or BDFP1.6-BV (as the acceptor) in order to increase the fluorescence brightness. As PBP fluorescent probes, this study achieved dual-color imaging with red light and far-red light for the first time [67]. Additionally, the method of fusion strategies to enhance cell brightness provides a new idea for developing more excellent fluorescent proteins. In another study, Zhao et al. [68] fused the red fluorescent protein mCherry with BDFP1.6 in different ways and obtained a novel fluorescent protein with a large Stokes shift (called BDFP2.0). BDFP2.0 can be excited with 580 nm excitation light and it has the characteristics of acid and alkali resistance, chemical denaturation resistance, and so on. It effectively strengthened the brightness in the far-red range through FRET. A mutant called ApcF2-12 with special spectral characteristics was obtained by modifying the ApcF2 subunit. Through mutating ApcF2-12, both brighter mutants and mutants with a special fluorescence spectrum were obtained. The mutants had large Stokes shifts and blue-shifted spectra. This work is a breakthrough in the PBP research which not only enriches the sources of mutants for screening excellent fluorescent probes but also provides new references and ideas for the development of fluorescent probes of PBPs.

## 5. Application of PBPs Fluorescent Probes

Owing to their exceptional autofluorescence properties, PBPs have been widely used in immunology, histochemistry, fluorescence spectroscopy, confocal microscopy, and flow cytometry in pharmacology and molecular biology [69]. The fluorescence spectrum demonstrates different fluorescence colors and emission wavelengths, including C-PE (yellow orange, 568–575 nm), C-PC (strong red, 630–650 nm), PEC (orange, 600–610 nm), R-PE (yellow orange, 570–575), R-PC (strong red, 635–638 nm), and APC (weak red, 660–665 nm) [15]. Likewise, PBPs have vibrant colors due to the intense fluorescence of PC, PE, and APC in cyanobacteria and red algae. Therefore, natural fluorescent proteins have broad application prospects in biotechnology, bioengineering, biomedicine, and other fields (Table 2).

### 5.1. Biological Detections

#### 5.1.1. Biomolecule Detection

Subunits of PC, PE, and APC are used as molecular probes to be labeled to antibodies, immunoglobulins, proteins, and affinins for imaging detection of biomolecules in cells [70]. Compared to PC and APC, PE is used more efficiently than other PBPs because it has an γ subunit which makes the fluorescent properties more stable [71]. PE integrated with Cy5/Cy7 dye was applied successfully to multicolor fluorescence for facilitating detection and visualization in molecular biology [72,73].

Ghosh et al. [74] created C-PE/graphene oxide composites to efficiently detect dsDNA in nanomolar quantities via a fluorescent “turn off/on” mechanism. Wu et al. [75] developed a simple, mix-and-detect fluorescent biosensor for DNA detection by exploiting the PDADMAC-mediated R-PE/BHQ2-ssDNA interaction. The biosensor exhibited good selectivity down to single-base mismatch and high sensitivity with a detection limit of 0.17 nM. Seyedi et al. [76] found that the drug transport processes can be monitored by the fluorescence signal of PC and HAS complexes. Also, bursal disease virus can be detected by an R-PE probe cross-linked to SPDP rapidly and accurately [77]. Recently, the recombinant streptavidin–PBP fusion protein was prepared and applied to the detection system of liquid phase microarray of pathogenic *Vibrio vulnificus* [47].

#### 5.1.2. Immunofluorescence Analysis

For immunofluorescence analysis, PBPs are also available as fluorescent markers for staining genomic DNA, nucleated cells, lymphocytes, red blood cells, white blood cells, and platelets [78]. Similarly, specific cells containing components of PBPs cross-linked with immunoglobulin were screened by flow cytometry for cell analysis. Neutrophils are important indicators in the immune system and can be used to detect neutropenia. Yan developed a clinically applicable method for the detection of anti-neutrophil antibodies by flow cytometric (FCM) for preliminary clinical application, where lymphocytes and granulocytes in whole blood samples were distinguished through forward and side scatter parameters; a granulocyte gate was set and the percentages of neutrophils expressing CDl6b and CDl77 antigen cells on the surface were detected by PE and APC monoclonal antibodies, respectively [79]. Telford et al. [80] demonstrated that APC, together with PBXL-3 and conjugated avidin molecule, was accessed as a fluorochrome for FCM immunodetection of surface antigens on immune cells. Interestingly, the conjugation of *Mannheimia haemolytica* with the R-PE can be phagocytized by neutrophils and macrophages in the blood. This is very beneficial for detection [81]. Wu et al. [39] reported a novel recombinant PBP fluorescent probe, a fusion protein (SLA) of core streptavidin from *Streptomyces avidinii* and ApcA, for the immunofluorescent detection of a tumor marker (α-fetoprotein, AFP). Similarly, Ge et al. [48] designed two dual-functional SA–PBPs (SA–PCA–PCB and SA–PCA–PCB) and evaluated their application for the detection of tumor markers (AFP and CEA). Both studies demonstrated the potential of recombinant PBPs in immunofluorescence analysis.

#### 5.1.3. Detection in Other Fields

Fluorescent probes for PBPs are being increasingly established for applications in environmental monitoring, in addition to the above-mentioned circumstances. PC fluorescent probes in the form of trimers are applied to the LED-CCD fluorescent density strip qualitative detection system. It provides convenient quantitative information and shows great potential for the rapid detection in environmental and food safety studies [9]. Bacteria-specific behaviors will be given because of the influence of different surface attachments in different growth environments [82]. Simis et al. [83] conducted experiments using single-cell Cyanobacteria isolated from Japanese waters and found that PC-based fluorescence monitoring of cyanobacterial complexes online is a fast, efficient, and versatile method for determining live cell concentrations. A positive correlation was found between PC fluorescence and cyanobacterial biomass within a certain concentration range [84]. Therefore, marine cyanobacterial populations can be monitored online by measuring intracellular PC content to provide a solution to water quality problems caused by the cyanobacterial bloom phenomenon.

### 5.2. Detection of Ions

PBPs can detect specific heavy ions by binding to certain substances as a labeling agent. In most cases, these heavy ions cause environmental pollution and are even harmful to human health. Therefore, it is important to detect the levels of these ions quickly and accurately.

Hou et al. [85] used CPC as a fluorescent probe to detect Hg^2+^ ions in seafood. It was found that Hg^2+^ ions triggered the CPC aggregation phenomenon, which could quench the fluorescence of CPC. The concentration of Hg^2+^ ions can be determined by monitoring the variation of fluorescence intensity. Similarly, Bhayani et al. [86] exploited the fluorescence quenching ability of CPC with heavy metals to establish a biosensor specifically for the detection of Hg^2+^ in low concentrations. Compared with 11 other different metal ions (Cr^3+^, Zn^2+^, Fe^2+^, Mg^2+^, Ni^2+^, Pb^2+^, Cu^2+^, Co^2+^, Ca^2+^, Ag^+^, and Li^+^), Hg^2+^ has a stronger binding to CPC indicated by the higher Gibbs free energy value. Thus, CPC could be further utilized as a sensitive biosensor for the detection of perilous Hg^2+^ in polluted water containing various metals. In addition, it can be used not only in the environment but also in food inspection in the future. Wei et al. [87] exploited C-PC as a protective agent to synthesize C-phycocyanin-Ag nanoparticles (PC-AgNPs). It was found that the PC-AgNPs may have a contributed to the detection of Cu^2+^ ions in diverse water bodies. Xu et al. [88] used R-PE-linked silver nanoparticles (AgNPs) to synthesize a novel probe, the R-PE-AgNPs probe. This probe has great potential for tracing Cu^2+^ ions in diverse aqueous media. PBPs show higher sensitivity and accuracy compared with traditional methods. The rapid detection of ions by fluorescent sensors using PBPs would be widely used in the environment, food, and other fields.

### 5.3. Fluorescent Image

In the visible region, some components of biological tissues are self-excited to produce autofluorescence, while the scattered light intensity of the sample is large, which seriously interferes with fluorescence detection and image. NIR/FR light detects samples with a high penetration, high imaging resolution, high detection sensitivity, and high signal-to-noise ratio. Recently, the development of BV-based PBPs probes has received more attention [24]. The BDFPs obtained by the molecular evolution of ApcF2 binds the more readily accessible BV covalently while retaining the red-shifted fluorescence in the near-infrared spectral region. They have been demonstrated as biomarkers in conventional and super-resolution microscopy as well as in several mammalian cells in vivo [89]. SmURFPs can incorporate a more membrane-permeant BV analog to improve membrane permeability, making smURFP fluorescence comparable to that of FPs from jellyfish or coral. A far-red and near-infrared fluorescent cell cycle indicator was created with smURFP and a BPH FP [64].

The use of photodynamic therapy and photothermal therapy with nanoparticles have attracted wide attention because of their therapeutic effects on cancer. Polypyrrole nanoparticles prepared from bovine serum albumin-phycocyanin complex were not only stable in various physiological solutions but also effectively killed MDA-MB-231 cells in a dual way under laser irradiation. In addition, it also produced ultrasonic signal amplitude, which was beneficial for imaging the treated cells [10]. Fluorescent proteins (FPs) imaging with red excitation light in the “optical window” above 600 nm is a potential method for the noninvasive visualization of gene-labeled cells which plays an important role in animal disease models. Chu et al. [90] found that mCardinal obtained by structure-guided mutagenesis of mNeptune can be used to non-invasively and vertically observe the differentiation of myoblasts from living mice, with high anatomical details.

**Table 2:** Applications of PBPs fluorescence probe.

Types	Fields of Application	Specific Example	Reference
APC	Immunofluorescence analysis	Flow cytometric detected anti-neutrophil antibodies.	[79]
		APC, together with PBXL-3 and conjugated avidin molecule accessed as a fluorochrome for FCM immunodetection of surface antigens on immune cells.	[80]
		Recombinant PBP fluorescent probe (SLA) detected AFP.	[39]
	Fluorescent image	BDFPs used as biomarkers in several mammalian cells in vivo.	[89]
		SmURFP and a BPH FP created a far-red and near-infrared fluorescent cell cycle indicator.	[64]
PC	Fluorescent image	Polypyrrole nanoparticles prepared from the albumin–phycocyanin complex killed MDA-MB-231 cells in a dual way under laser irradiation.	[10]
	Biomolecule detection	PC and HAS complexes monitored drug transport processes.	[76]
	Immunofluorescence analysis	Two dual-functional SA–PBPs (SA–PCA–PCB and SA–PCA–PCB) detected AFP and CEA.	[48]
	Other	PC trimers applied to the LED-CCD fluorescent density strip qualitative detection system.	[9]
		PC-based fluorescence monitoring of cyanobacterial complexes online determined live cell concentrations	[83]
	Detection of ions	CPC fluorescent probe detected Hg^2+^ ion in seafood.	[85]
		CPC biosensor detected Hg^2+^ in low concentrations.	[86]
		PC-AgNPs detected Cu^2+^ ions in diverse water bodies.	[87]
PE	Biomolecule detection	Using liquid phase microarray detect vibrio combined with recombinant streptavidin-phycoerythrin specifically.	[47]
		PE integrated with Cy5/Cy7 dye facilitated detection and visualization in molecular biology.	[72,73]
		C-PE/graphene oxide composites detect dsDNA in nanomolar quantities.	[74]
		Using the PDADMAC-mediated R-PE/BHQ2-ssDNA interaction, the fluorescent biosensor detected the target DNA.	[75]
		R-PE probe cross-linked SPDP detected bursal disease virus.	[77]
	Immunofluorescence analysis	Flow cytometric detected anti-neutrophil antibodies.	[79]
		R-PE labeled *Mannheimia haemolytica* can be monitored by observing fluorescence.	[81]
	Detection of ions	R-PE-AgNPs traced Cu^2+^ ions in diverse aqueous media.	[88]

## 6. Conclusions and Perspective

PBPs are a large family of light-harvesting biliproteins found in cyanobacteria, cryptomonads, and red algae. Due to their exceptional spectral properties, natural PBPs have been widely used in immunology, histochemistry, confocal microscopy, flow cytometry, and immunofluorescence analysis for many years. The purification of natural PBPs is generally complex which requires multiple steps for high PBPs purification. In some circumstances, natural PBPs may cause steric hindrance in immunofluorescence analysis due to their large molecular weights. Biosynthesis of recombinant PBPs by engineered microorganisms would offer an attractive strategy for PBP preparation. The cultivation of microorganisms is easily scaled up and the recombinant PBPs can be purified by one-step affinity chromatography. The simple and efficient preparation of recombinant PBPs makes it possible to prepare a large number of PBPs at a low cost. Moreover, recombinant subunits of PBP with relatively smaller sizes would be helpful to overcome the steric hindrance in cell sorting and immunofluorescence analysis. In particular, FPs evolved from PBP absorb light and emit fluorescence in the NIR/FR region, making engineered PBP a valuable fluorescent probe for cell imaging. These advances in recent years demonstrate that novel fluorescent proteins can be obtained through gene engineering and protein engineering. In this respect, it is critical to resolve crystal structures with high resolution and elucidate the relationship of fluorescent properties and their structure, which will help the creation of a series of novel PBPs with distinct absorption and fluorescent spectra and high absorption coefficients and quantum yield.

As research on PBPs continues, the use of new tags and the discovery of effective labeling modalities (e.g., mechanisms of interactions between PBPs and specific molecules) is likely to serve as a popular area in the future, even if these tags bring about positive or negative effects on recombinant PBPs.

## Figures and Tables

**Table 1:** Production of recombinant apo- or holo-phycobiliproteins.

PBPs	Sources for PBP Gene	Hosts	Phycobilins	Lyases
Apo-ApcA, Apo-ApcB	*Synechococcos* sp. PCC 7002	*E. coli*	PCB	-
Apo-APC	*Asterocapsa nidulans* UTEX 625	*E. coli*	PCB	-
Apo-CpcA	*Asterocapsa nidulans* R2	*E. coli*	PCB	-
Holo-CpcA	*Synechocystis* sp. PCC 6803	*E. coli*	PCB	CpcE/CpcF
Holo-PecA	*Anabaena* sp. PCC 7120	*E. coli*	PVB	PecE/PecF
Holo-ApcAB	*Synechococcus* sp. PCC 7002	*E. coli*	PCB	CpcU/CpcS
Holo-ApcAB	*Synechocystis* sp. PCC 6803	*E. coli*	PCB	CpcU/CpcS
Holo-ApcAB	*Gracilaria chilensis*	*E. coli*	PCB	CpcU/CpcS
Holo-ApcA	*Synechococcus elongatus* BP-1	*E. coli*	PCB	CpcS
Holo-ApcB	*Spirulina* sp.	*E. coli*	PCB	CpcS
Holo-ApcA	*Synechococcus elongatus* BP-1	*E. coli*	PCB	CpcS
Streptavidin-Holo-ApcA	*Synechococcus elongatus* BP-1	*E. coli*	PCB or PEB	CpcS
Streptavidin-Holo-ApcA	*Synechococcus elongatus* BP-1	*E. coli*	PEB	CpcS
Holo-ApcB	*Synechococcus elongatus* BP-1	*E. coli*	PCB	CpcS
Holo-ApcF	*Synechococcus* sp. PCC 7002	*E. coli*	PCB	CpcU/CpcS
Holo-CpcA	*Synechocystis* sp. PCC 6803	*E. coli*	PCB	CpcE/CpcF
Holo-CpcB	*Synechocystis* sp. PCC 6803	*E. coli*	PCB	CpcU/CpcS
Holo-CpcB	*Synechocystis* sp. PCC 6803	*E. coli*	PCB	CpcT
Holo-CpcB	*Synechococcus elongatus* BP-1	*E. coli*	PEB, PUB	CpcU, CpcT
Holo-CpcA	*Synechocystis* sp. PCC 6803 *Synechococcus* sp. PCC 7002	*E. coli*	PCB, PEB, PΦB, PUB, PVB, PtVB	CpcE/CpcF PecE/PecF
Holo-CpeA	*Microchaete diplosiphon* UTEX481	*E. coli*	PEB	CpeY
Holo-CpeB	*Synechococcus* sp. RS9916	*E. coli*	PUB	MpeV
Holo-CpeB	*Microchaete diplosiphon*	*E. coli*	PEB	CpeF
Holo-CpeB	*Prochlorococcus marinus* MED4	*E. coli*	PEB	CpeS
PcA/PcB	*Gracilariopsis lemaneiformis*	*E. coli*	PCB	CpcU/CpcS, CpcE/CpcF, CpcT
Holo-MpeA	*Synechococcus* sp. RS9916	*E. coli*	PUB	MpeZ
Holo-C-PC equipped with different tags	*Anabaena* sp. PCC7120	*Anabaena* sp. PCC 7120	PCB	-
Holo-APC	*Cyanophora paradoxa* (Glaucophyta)	*Synechococcus* sp. PCC 7002	PCB	-

## Data Availability

Data will be made available upon request.

## Abbreviations

PBP, phycobiliprotein; APC, allophycocyanin; PC, phycocyanin; PE, phycoerythrin; PCB, phycocyanobilin; PUB, phycourobilin; PEB, phycoerythrobilin; PXB, phycoviolobilin; BPH, bacteriophytochrome; DTT, dithiothreitol; Cy5, cyanine 5; Cy7, cyanine 7; PMT, photomultiplier; APD, Avalanche Photodiode; SMCC, succinimidyl trans-4-(maleimidylmethyl) cyclohexane-1-carboxylate; SPDP, succinimidyl 3-(2-pyridyldithio) propionate; BV, biliverdin; HO1, heme oxygenase; SDS-PAGE, sodium dodecyl sulfate-polyacrylamide gel electrophoresis; AFP, α-fetoprotein; CEA, carcinoembryonic antigen; ELISA, enzyme linked immunosorbent assay; FPs, fluorescent proteins; H_2_O_2_, hydrogen peroxide; SmURFP, small ultra-red fluorescent proteins; Tandem dimer smURFP, TDsmURFP; BDFPs, BeiDou Fluorescent Proteins; PФB, phytochromobillin; FCM, flow cytometric.
